# Performance of electrophysiologic study in an asymptomatic patient with type 2 intermittent Brugada syndrome: To do or not to do

**DOI:** 10.22088/cjim.9.1.92

**Published:** 2018

**Authors:** Kaveh Hosseini, Mansour Jahangiri, Ali Vasheghani Farahani

**Affiliations:** 1 Tehran Heart Center, Tehran University of Medical Sciences, Tehran, Iran

**Keywords:** Brugada syndrome, Supraventricular Tachycardia, Electrophysiology study.

## Abstract

**Background:**

Brugada syndrome (BrS) is an inherited channelopathy, which is associated with sudden cardiac death due to rapid polymorphic VT or VF. There is no definite consensus regarding the management of asymptomatic patients. Some experts advocate close follow-up; others propose the programmed stimulation for risk stratification. We aimed to evaluate the benefit of complete atrial and ventricular stimulation in patients with BrS and palpitation.

**Case Presentation:**

A 30-year-old man was admitted to our hospital because of a family history of sudden cardiac death (SCD) at age less than 45 years. He complained of self-terminated episodes of palpitation with no history of syncope. Baseline ECG showed incomplete right bundle branch block (RBBB) and saddle-back-like ST deviation in V1. Flecainide challenge test (FCT) revealed Brugada pattern. Complete EPS was done for evaluation of VT/VF inducibility and probable concomitant supraventricular arrhythmias.

Programmed atrial stimulation showed inducible typical slow-fast AVNRT with AH jump 75 msec. Successful slow pathway ablation was done. There was no inducible ventricular arrhythmia.

**Conclusions:**

Patients with drug-induced BrS, positive family history of SCD and also episodes of palpitation, benefit from complete EPS. However, ICD implementation is not recommended in asymptomatic patients with drug-induced BrS and negative EPS for ventricular stimulation.

Brugada syndrome (BrS) is an autosomal dominant disorder characterized by ST segment elevation in right precordial leads and an increased risk of sudden cardiac death (SCD) (1). The prevalence of Brugada ECG is higher in Asia than in the United States and Europe (2). The main cause of sudden death in this syndrome is ventricular fibrillation (VF). There is not definitive treatment modality that reliably and totally prevents ventricular fibrillation in this syndrome. Prevention of this lethal arrhythmia before it causes SCD is cardinal. Guideline-based implementation of an implementable cardioverter defibrillator (ICD) is recommended in selected cases^3^. In this case report, an asymptomatic patient with Brugada syndrome was admitted due to self-limited palpitation and was evaluated for malignant ventricular arrhythmias. 

## Case Presentation

A 30-year-old man was admitted to our hospital because of a family history of sudden cardiac death in two uncles and one cousin, and suspicious ECG pattern, in favor of BrS. He also complained of frequent self-terminated episodes of palpitation in the past 3 years. Frequency and duration of palpitation attacks increased in the past 4 months.

**Figure 1:**
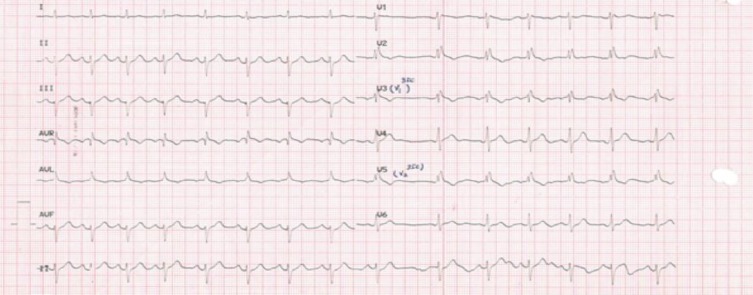
Baseline surface ECG.

He took propranolol whenever the attacks started, but as he described; ‘’nothing just happens with this pill!’’. There was no episode of syncope, loss of conscious in his lifetime. Physical examination and lab results were normal. Both coronary angiogram and echocardiography revealed no structural heart disease. 24-hour holter monitoring was unremarkable. Baseline ECG showed incomplete RBBB and saddle-back-like ST deviation in V1. Figure1.

**Figure 2:**
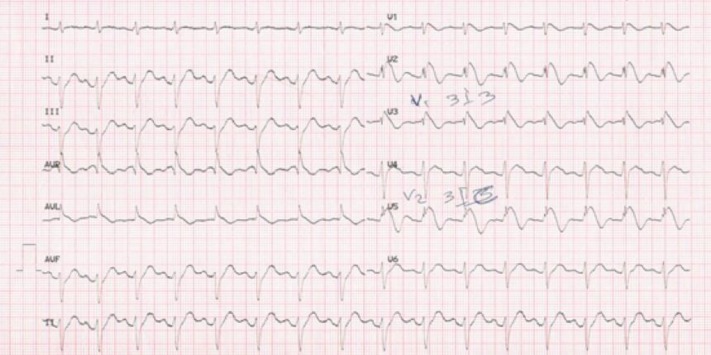
Surface ECG after Flecainide Challenge Test

To confirm the diagnosis, the patient underwent Flecainide challenge test (FCT) in coronary care unit. After informed consent from the patient, 400 mg of oral Flecainide was used to perform the test.12-lead ECG was recorded every 30 min for 3 hours, and the patient was under precise cardiac monitoring for 6 hours. Type-2 BrS was manifested 1 hour after the test, (figure 2). The patient had no complaint during FCT. 

These changes disappeared 5 hours after the beginning of the test and reappeared spontaneously in tomorrow morning. Hence, an intermittent form of BrS was suspected in this patient.

**Figure 3:**
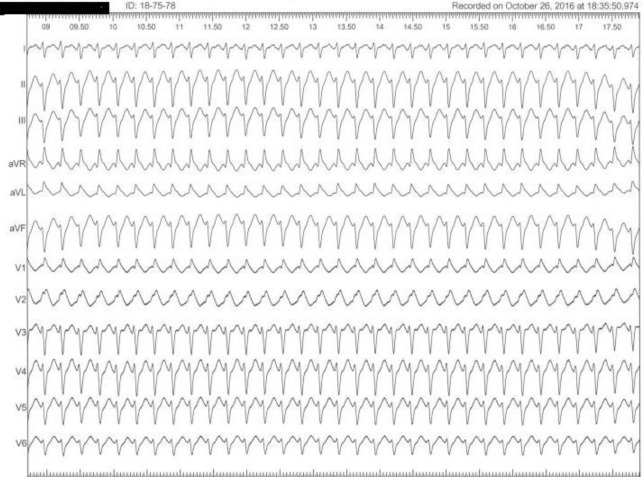
Surface ECG during episode of palpitation

**Figure 4:**
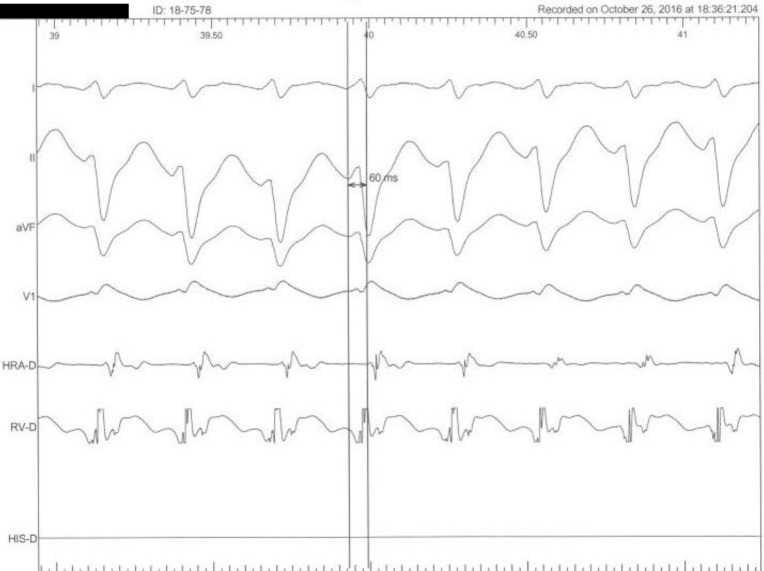
EP tracing. Narrow complex Short RP tachycardia

Based on history of palpitation and also family history of SCD, the patient underwent complete EPS. There was no inducible VF or VT in ventricular stimulation. However, programmed atrial stimulation showed inducible typical slow-fast AVNRT with AH jump 75 msec. Successful slow pathway ablation was done (figures 3 and 4). 

## Discussion

SCD is attributed to dangerous ventricular arrhythmia in these patients. Based on accepted guidelines, ICD implementation in symptomatic BrS who are survivors of an aborted cardiac arrest and/or have documented spontaneous sustained VT is recommended and should be considered in patients with a spontaneous diagnostic type I ECG pattern and history of syncope. The question about the role of complete EPS in newly diagnosed BrS is still up for discussion.

The association between BrS and supraventricular arrhythmias was first described in 2001 (4). Dysfunction of sodium channel that causes malignant arrhythmias in BrS, is attributed to mutation of SCNA5 gene (5). In recent studies, the idea about extension of this and other sodium channel mutations to atrial myocardium and concomitant supraventricular re-entry type arrhythmias, is on the table nowadays (6, 7). Patients with BrS may experience benign episodes of self-terminated palpitation without syncope or aborted SCD. Idiopathic VF and SVT may both be present in BrS. 

Routine EPS as a tool for risk stratification in asymptomatic patients is still a challenging subject. The second consensus report^8^ recommends EPS in asymptomatic patients with spontaneous type 1 ECG and ICD implantation be recommended if the EPS is positive (class 2A) and a close follow up, if the EPS is negative. If the patient is asymptomatic and BrS ECG changes appeared just after a drug challenge, ICD implantation is indicated only if EPS is positive (class 2B) (3).

There is no consensus on the value of the EPS in predicting outcome. While Brugada et al. considered sustained ventricular arrhythmia as a strong predictor of SCD (9) while others do not. The PRELUDE (programmed electrical stimulation predictive value) (10) registry failed to support the view that lack of indelibility has negative predictive value in BrS. The FINGER (France, Italy, Netherlands, Germany) registry (11), found that inducibility of sustained ventricular arrhythmias was significantly associated with a shorter time to first arrhythmic event in the univariate analysis, but in the multivariate analysis, it did not predict future arrhythmic events.

Our patient was asymptomatic and ECG changes were evident after Flecainide challenge test (FCT). According to the guidelines of the Cardiac Society of Australia and New Zealand (3), EPS is indicated (class 2B), and in the case of positive EPS, ICD implementation should be considered. We performed EPS; no ventricular arrhythmia was induced, hence, we did not recommend ICD to the patient. On the other hand, based on aforementioned guidelines, EPS is recommended for investigation of associated SVT. Chief complaint of bothering palpitation in this case, made us to perform a complete EPS. So, one may conclude that EPS is logical when positive FCT and/or history of palpitation are present in a patient with BrS. The latest consensus on BrS in 2015 (12), emphasized on EPS in asymptomatic patients with positive family history, but data about patients with negative family history is still lacking (13). These guidelines also recommend ICD for patients with all these three criteria; 1) spontaneous or drug-induced type 1 BrS, 2) Positive family history of SCD and 3) inducible VF/VT in EPS. 

In conclusion, in this case, based on his positive family history of SCD and also episodes of palpitation, he benefits from complete EPS. Since, he did not fulfill all the branches of the above triad; he is not a good candidate for ICD implementation.

## References

[1] Napolitano C, Priori SG (2006). Brugada syndrome. Orphanet J Rare Dis.

[2] Mizusawa Y, Wilde AA (2012). Brugada syndrome. Circ Arrhythm Electrophysiol.

[3] Vohra J, Rajagopalan S (2015). Update on the diagnosis and management of Brugada syndrome. Heart Lung Circ.

[4] Eckardt L, Kirchhof P, Loh P (2001). Brugada syndrome and supraventricular tachyarrhythmias: a novel association?. J Cardiovasc Electrophysiol.

[5] Wang DW, Makita N, Kitabatake A, Balser JR, George AL Jr (2000). Enhanced Na+ channel intermediate inactivation in Brugada syndrome. Circ Res.

[6] Liu B, Guo C, Fang D, Guo J (2015). Clinical observations of supraventricular arrhythmias in patients with brugada syndrome. Int J Clin Exp Med.

[7] Hasdemir C, Payzin S, Kocabas U (2015). High prevalence of concealed Brugada syndrome in patients with atrioventricular nodal reentrant tachycardia. Heart Rhythm.

[8] Antzelevich C, Brugada P, Borggrefe M (2005). Brugada syndrome. report of the second consensus conference. ACC Curr J Rev.

[9] Brugada P, Brugada R, Brugada J (2005). Patients with an asymptomatic Brugada electrocardiogram should undergo pharmacological and electrophysiological testing. Circulation.

[10] Priori SG, Gasparini M, Napolitano C (2012). Risk stratification in Brugada syndrome: results of the PRELUDE (PRogrammed ELectrical stimUlation preDictive valuE) registry. J Am Coll Cardiol.

[11] Probst V, Veltmann C, Eckardt L (2010). Long-term prognosis of patients diagnosed with Brugada syndrome results from the FINGER Brugada Syndrome Registry. Circulation.

[12] Ackerman MJ, DeSimone CV (2015). Programmed electrical stimulation for patients with asymptomatic brugada syndrome?: the shock-filled debate continues. J Am Coll Cardiol.

[13] Wilde AA, Antzelevitch C, Borggrefe M (2002). Proposed diagnostic criteria for the Brugada syndrome consensus report. Circulation.

